# Platelet‐to‐lymphocyte ratio could be a promising prognostic biomarker for survival of colorectal cancer: a systematic review and meta‐analysis

**DOI:** 10.1002/2211-5463.12083

**Published:** 2016-06-16

**Authors:** Hong‐Xin Peng, Kang Lin, Bang‐Shun He, Yu‐Qin Pan, Hou‐Qun Ying, Xiu‐Xiu Hu, Tao Xu, Shu‐Kui Wang

**Affiliations:** ^1^ Medical School of Southeast UniversityNanjingJiangsuChina; ^2^ Central LaboratoryNanjing First HospitalNanjing Medical UniversityNanjingJiangsuChina

**Keywords:** colorectal cancer, meta‐analysis, platelet‐to‐lymphocyte ratio, prognosis

## Abstract

Inflammation is one of the most important causes leading to colorectal carcinogenesis, and inflammatory biomarkers such as the platelet‐to‐lymphocyte ratio (PLR) might predict survival in colorectal cancer (CRC). However, the prognostic value of PLR in CRC patients remains controversial. The prognostic value of PLR was comprehensively analyzed in 12 articles including 3541 CRC patients (10 for overall survival (OS), seven for disease‐free survival (DFS), three for recurrence‐free survival (RFS), and three for cancer‐specific survival (CSS)) in this study. The overall pooled hazard ratios (HRs) of PLR for OS, DFS, and CSS were significant at 1.29 (95% confidence interval, CI = 1.13–1.47, *P*_H_ = 0.149), 1.43 (95% CI = 1.03–1.97, *P*_H_ = 0.025), and 1.26 (95% CI = 1.04–1.52, *P*_H_ = 0.223), respectively. However, there was no evidence of significance for RFS (HR = 1.29, 95% CI = 0.98–1.70, *P*_H_ = 0.231) in our study. Stratified analyses indicated elevated PLR was a predictor of poor OS (metastatic patients) and DFS (Caucasian population) and was also significantly associated with OS in univariate analysis (HR = 1.35, 95% CI = 1.14–1.60, *P*_H_ = 0.532) and those only treated surgically (HR = 1.37, 95% CI = 1.10–1.70, *P*_H_ = 1.080). However, our findings indicated that elevated PLR is a promising prognostic biomarker for colorectal cancer, especially in metastatic Caucasian CRC patients.

**Abbreviations**
95% CI: 95% confidence interval
CRC: colorectal cancer
CRM: cancer‐related mortality
CRP: C‐reactive protein
CSS: cancer‐specific survival
DFS: disease‐free survival
HR: hazard ratio
NLR: neutrophil‐to‐lymphocyte ratio
OS: overall survival
PFS: progression‐free survival
*P*_H_: *P*‐value of heterogeneity
PLR: platelet‐to‐lymphocyte ratio
RFS: recurrence‐free survival
TTR: time to recurrence

Colorectal cancer (CRC) is the third most common cancer and the fourth leading cause of cancer‐related death worldwide 1. In 2011, approximately 310 244 newly diagnosed cases and 149 722 CRC‐related deaths were reported in 2015 China cancer registry annual report, which accounted for 20% and 25% of the total in the world, respectively 2. Nowadays, obvious improvements are developed and applied in diagnosis and treatment for CRC; however, due to the local tumor recurrence or metastasis, 5‐year survival of the patients is still not promising. Thus, identification of effective early diagnostic, treatment predicting, and prognostic biomarkers are essential for survival improvement of CRC individuals.

Inflammation is one of the most important causes leading to CRC. Cancer‐related inflammation could aid malignant cell in the proliferation, infiltration, metastasis, regulating the innate and adaptive immune responses, and affecting the drug effect 3. Numerous studies have demonstrated that systemic inflammatory response counted for the development and progression of various cancers, including CRC 3, 4, 5. Systemic inflammatory state could be measured by many biomarkers, such as the albumin, C‐reactive protein (CRP), serum procalcitonin, cytokines, leukocyte and its subsets 6, 7, 8, neutrophil‐to‐lymphocyte ratio (NLR), and platelet‐to‐lymphocyte ratio (PLR). CRP, albumin, serum procalcitonin, and cytokines costed a lot and their prognostic values were finite 7, and elevated NLR had been verified to be a poor prognostic biomarker for many solid tumor 9, 10, 11, 12, including CRC. PLR (platelet count divided by lymphocyte count), cheap and available, also was regarded as a high efficient prognostic biomarker, for many tumors 13, 14, 15, 16. However, the relationship of PLR in CRC was still at loggerheads. Some studies reported that elevated PLR could be considered as a prognostic biomarker for CRC 17, 18, 19, 20, 21, 22, 23, yet others showed that PLR was not associated with the clinical outcome of CRC 24, 25, 26, 27.

Therefore, in this study, a meta‐analysis with 12 articles including 3541 CRC patients was conducted to comprehensively analyze the relationship of PLR and CRC survival, and investigate whether PLR could be a promising prognostic biomarker for CRC.

## Materials and methods

### Search strategy

The relative literature was searched in PubMed and Web of Science database in accordance with following keywords: ‘PLR’ OR ‘platelet lymphocyte ratio’ OR ‘platelet to lymphocyte ratio’ OR ‘platelet‐lymphocyte ratio’ OR ‘platelet‐to‐lymphocyte ratio’ AND ‘CRC’ OR ‘colorectal cancer’ OR ‘colorectal carcinogenesis’ OR ‘colorectal tumor’ OR ‘colorectal neoplasm’ from October 2000 to October 2015. Meanwhile, relative studies were also screened by manual retrieving the reference list of relative literature.

### Inclusion and exclusion criteria

The eligible study was included when: (a) it published in the form of original article in English; (b) correlation of PLR with survival was reported; (c) CRC was diagnosed according to histopathological examination. Also, letter, conference abstract, review article, duplicated study, and study failed to present cut‐off value of PLR or hazard ratio (HR) and its 95% confidence interval (CI) were excluded from the study.

### Data extraction

According to preferred reporting items for systematic reviews and meta‐analyses (PRISMA) statement and methods 28, 29, two researchers (HXP and KL) screened and assessed the articles independently in accordance with inclusion and exclusion criteria and collected information using predesigned forms. The following clinical characteristics were extracted: first author of the study, year of publication, number of patients, median age, country, ethnicity, TNM stage, methods of treatment, follow‐up time of enrolled patients, cut‐off value of PLR, analysis method, and HR with its 95%CI. Overall survival (OS) and disease‐free survival (DFS) were regarded as a master outcome of interest, and others were treated as the secondary outcomes. In addition, only if multivariate analysis was not available could univariate analysis be used. Any conflicts were solved by discussion or decision by the third reviewer (HQY) before analysis.

### Statistical analysis

Pooled HR and 95% CI were used as common measurements for assessing the strength between pretreatment PLR and survival of CRC. Cochrane *Q* test and Higgins I‐squared statistics were performed to assess the heterogeneity of pooled studies. I‐square > 50% and *P*_H_ < 0.1 were considered as a measure of substantial heterogeneity among studies, then random‐effects model (DerSimonian–Laird method) 30 was used to calculate the pooled HR. Otherwise, fixed effects model (Mantel–Haenszel method) 31 was performed. Subgroup analysis was conducted to explore the sources of heterogeneity. Publication bias was assessed by Begg's funnel plot and Egger's linear regression test 32. The sensitivity analysis was performed to estimate the stability of outcome. All analyses were carried out by stata 11.0 statistical software (STATA Corporation, College Station, TX, USA) and *P* < 0.05 was considered statistically significant.

## Results

### Eligible article

**Figure 1:**
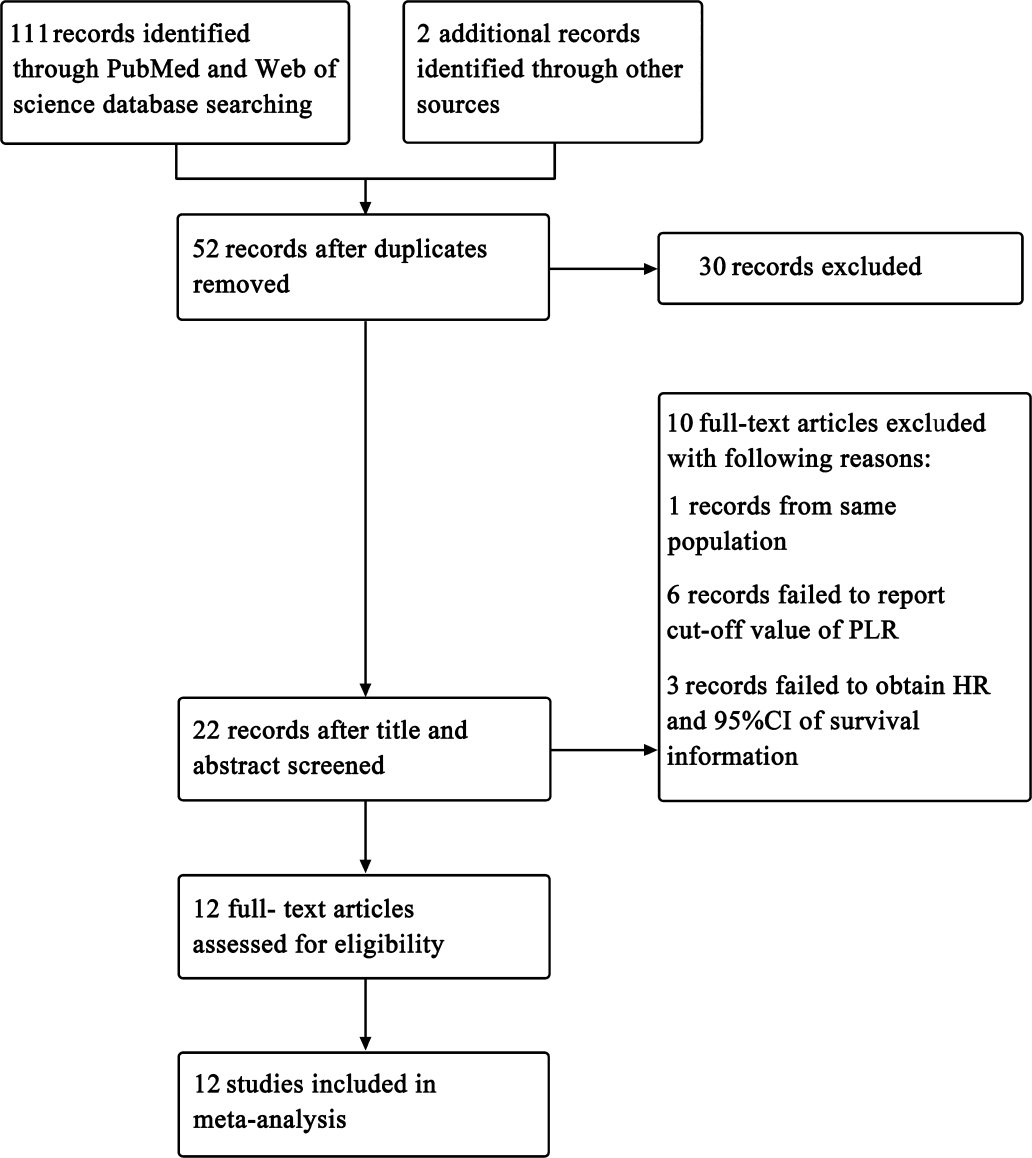
Selection of studies included in meta‐analysis.

According to the search strategy mentioned above, a total of 113 articles were identified thoroughly. After removing the duplicates, 52 records were retrieved. However, 30 records were excluded because of the title and abstract irrelevance of the inclusion criterion. After perusing the full text of the remaining 22 studies, 10 records were excluded for the following reasons: one study was from same population and nine studies failed to obtain relevant information such as survival information or cut‐off value of PLR. Finally, 12 studies 17, 18, 19, 20, 21, 22, 23, 24, 25, 26, 27, 33 including 3541 patients were included for this meta‐analysis (Fig. 1).

Characteristics of included studies are shown in Table 1; all of included studies were published in 2012 or later, five of them were reported in Asian population, and others were all Caucasian population. There were 10 for overall survival (OS), seven for disease‐free survival (DFS), three for recurrence‐free survival (RFS), three for cancer‐specific survival (CSS), one for cancer‐related mortality (CRM), and one for time to recurrence (TTR) in the eligible studies.

**Table 1:** Main characteristics of included studies

Study	Year	Number	Age (median)	Treatment	Follow‐up (month)	Sex (male/female)	TNM stage (I/II/III)	Ethnicity	Metastasis	Country	Survival
Choi 17	2015	549	68.7	Operation	NA	296/253	146/216/185	Caucasian	N	Canada	OS
Mori 18	2015	157	67	Operation	20.5 (0.2–62.4)	87/65	45/55/52	Asian	N	Japan	DFS
Neal 19	2015	302	66	Metastasectomy	29.7 (4–96)	192/110	–	Caucasian	Y	New York	OS/CSS
Neofyto 20	2014	140	NA	Hepatectomy	33 (1–103)	88/52	–	Caucasian	Y	London	OS/DFS
Azab 24	2014	380	69	Mix^a^	40.5	273/307	132/164/136	Caucasian	Y	USA	OS/DFS/CRM
Sun 25	2014	255	59.47 ± 12.63	Operation	NA	135/120	29/139/87	Asian	N	China	OS/DFS
Son 26	2013	624	NA	Operation	42.0 (1–66)	368/256	79/233/312	Asian	N	Korea	OS/DFS
Carruthers 27	2012	115	63.8	Chemoradiation	37.1	75/40	12/57/55	Caucasian	Y	UK	OS/DFS/RFS
Szkandera 33	2014	372	64	Operation	68	217/155	0/154/217	Caucasian	N	Austria	OS/TTR
Kwon 21	2012	200	64 ± 11.7	Operation	33.6	123/77	13/91/8	Asian	Y	Korea	OS
Ozawa 22	2015	234	NA	Operation	64 (1–173)	142/92	0/234/0	Asian	N	Japan	DFS/CSS
Ying 23	2014	205	NA	Operation	26 (14.5–60)	144/61	–	Asian	N	China	OS
Ying 23	2014	205	NA	Operation	19 (9–30)	144/61	–	Asian	N	China	CSS/RFS

a Mix means operation for stage I–III patients and chemoradiation for stage IV patients. NA, not available; OS, overall survival; DFS, disease‐free survival; RFS, recurrence‐free survival; CSS, cancer‐specific survival; CRM, cancer‐related mortality; TTR, time to recurrence; N, no; Y, yes.

### OS and PLR

**Figure 2:**
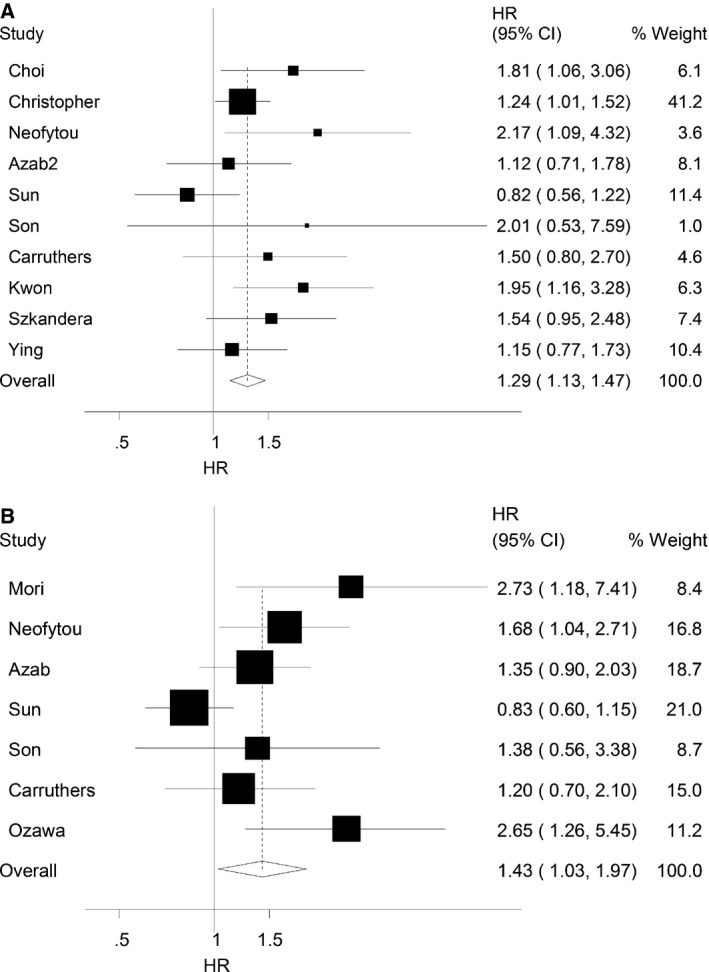
Forest plots showing the results of studies on the association between elevated PLR and prognostic outcome. (A) OS (according to fixed effect model); (B) DFS (according to random effect model).

There were 10 studies containing 3150 CRC patients reporting hazard ratios for OS and the main results are described in Table 2 and Fig. 2. Elevated PLR was significantly associated with a poor OS (HR = 1.29, 95% CI = 1.13–1.47, *P*_H_ = 0.149) in overall population. The stratified analyses showed that increased PLR was strongly associated with poor outcome in metastatic patients (HR = 1.32, 95% CI = 1.10–1.59, *P*_H_ = 0.287), Caucasian population (HR = 1.34, 95% CI = 1.14–1.58, *P*_H_ = 0.338), univariate analysis (HR = 1.35, 95% CI = 1.14–1.60, *P*_H_ = 0.532), and surgery only (HR = 1.37, 95% CI = 1.10–1.70, PH = 1.080) subgroups. However, we did not observe the significant association between PLR and OS in nonmetastatic patients (HR = 1.35, 95% CI = 0.97–1.86, *P*_H_ = 0.041), mixed group patients (HR = 1.19, 95% CI = 0.77–1.84, *P*_H_ = 0.417), Asian population (HR = 1.28, 95% CI = 0.90–1.80, *P*_H_ = 0.088), multivariate analysis (HR = 1.30, 95% CI = 0.95–1.79, *P*_H_ = 0.062), and treatments in addition to surgery (HR = 1.25, 95%CI = 0.86–1.80, *P*_H_ = 0.453) subgroups.

**Table 2:** The main results of pooled studies

Survival	Variables	No. of studies	No. of patients	*P‐*value			Regression model	
				*P*_H_	*P*_Z_	*P*_E_	Random	Fixed
OS	All	10	3150	0.149	0.001	0.162	1.33 (1.12–1.59)	**1.29 (1.13–1.47)** *
	Metastatic							
	YES	3	557	0.287	0.017	–	1.38 (1.06–1.80)	**1.32** (**1.10–1.59**)*
	NO	5	1581	0.041	0.073	–	**1.35 (0.97–1.86**)	1.28 (1.05–1.57)
	MIX	2	1012	0.417	0.429	–	1.19 (0.77–1.84)	**1.19 (0.77–1.84)**
	Ethnicity							
	Asian	5	1751	0.088	0.074	–	**1.28 (0.90–1.80)**	1.20 (0.96–1.50)
	Caucasian	5	1399	0.338	0.006	–	1.37 (1.14–1.65)	**1.34 (1.14–1.58)** *
	Analysis method							
	Univariable	4	1338	0.532	0.001	–	1.35 (1.14–1.60)	**1.35 (1.14–1.60)** *
	Multivarible	6	1812	0.062	0.103	–	**1.30 (0.95–1.79)**	1.30 (0.95–1.79)
	Treatment							
	Operation+	8	2647	0.080	0.005	–	**1.37 (1.10–1.70)** *	1.30 (1.13–1.49)
	Other+	2	503	0.453	0.241	–	1.25 (0.86–1.80)	**1.25 (0.86–1.80)**
DFS	All	7	1913	0.025	0.031	0.044	**1.43 (1.03–1.97)** *	1.26 (1.04–1.52)
	Metastatic							
	YES	2	255	0.365	0.043	–	1.45 (1.01–2.08)	**1.45 (1.01–2.08)** *
	NO	3	646	0.002	0.25	–	**1.71 (0.69–4.24)**	1.11 (0.84–1.47)
	MIX	2	1012	0.969	0.108	–	1.36 (0.94–1.96)	**1.36 (0.94–1.96)**
	Ethnicity							
	Asian	3	1113	0.015	0.406	–	**1.38 (0.65–2.92)**	1.04 (0.79–1.38)
	Caucasian	4	800	0.435	0.003	–	1.48 (1.14–1.92)	**1.48 (1.14–1.92)** *
	Analysis method							
	Univariable	2	272	0.132	0.102	–	1.66 (0.76–3.65)	**1.49 (0.93–2.39)**
	Multivarible	5	1641	0.021	0.027	–	**1.38 (0.94–2.04)**	1.22 (0.99–1.50)
	Treatment							
	Operation+	5	1410	0.006	0.079	–	**1.58 (0.95–2.64)**	1.24 (0.98–1.57)
	Other+	2	503	0.736	0.121	–	1.30 (0.93–1.80)	**1.30 (0.93–1.80)**
RFS	All	3	869	0.231	0.179	–	1.27 (0.90–1.80)	**1.29 (0.98–1.70)**
CSS	All	3	741	0.223	0.102	–	1.29 (0.95–1.75)	**1.26 (1.04–1.52)** *

The bold and “*” represent that HR with 95% CI was used to analyze and was statistically significant results, respectively. “+” “operation” group means patients who underwent surgery alone, and “other” group means patients who underwent metastasectomy or preoperative chemoradiation. *P*_H_, *P*‐value of heterogeneity test; *P*_Z_, *P*‐value of *t*‐test; *P*_E_, *P*‐value of Egger's test; OS, overall survival; DFS, disease‐free survival; RFS, recurrence‐free survival; CSS, cancer‐specific survival.

### DFS and PLR

Seven studies containing 1913 CRC patients were included to evaluate the association between PLR and DFS in CRC patients in this study. The pooled results showed that elevated PLR was associated with a poor clinical outcome for DFS (HR = 1.43, 95% CI = 1.03–1.97, *P*_H_ = 0.025). Stratifying overall population based on disease stage, ethnicity, analysis method, and treatment, PLR was only associated with the outcome of CRC among metastatic patients (HR = 1.45, 95% CI = 1.01–2.08, *P*_H_ = 0.365) and Caucasian (HR = 1.48, 95% CI = 1.14–1.92, *P*_H_ = 0.435) (Table 2).

### RFS, CSS, and PLR

The significant association was observed between CSS and PLR (HR = 1.26, 95% CI = 1.04–1.52, *P*_H_ = 0.223) in combination with three studies containing 741 CRC patients, whereas no significant association between RFS and PLR (HR = 1.29, 95%CI = 0.98–1.70, *P*_H_ = 0.231) was observed in combination with three studies including 869 CRC patients.

### Sensitivity analysis

**Figure 3:**
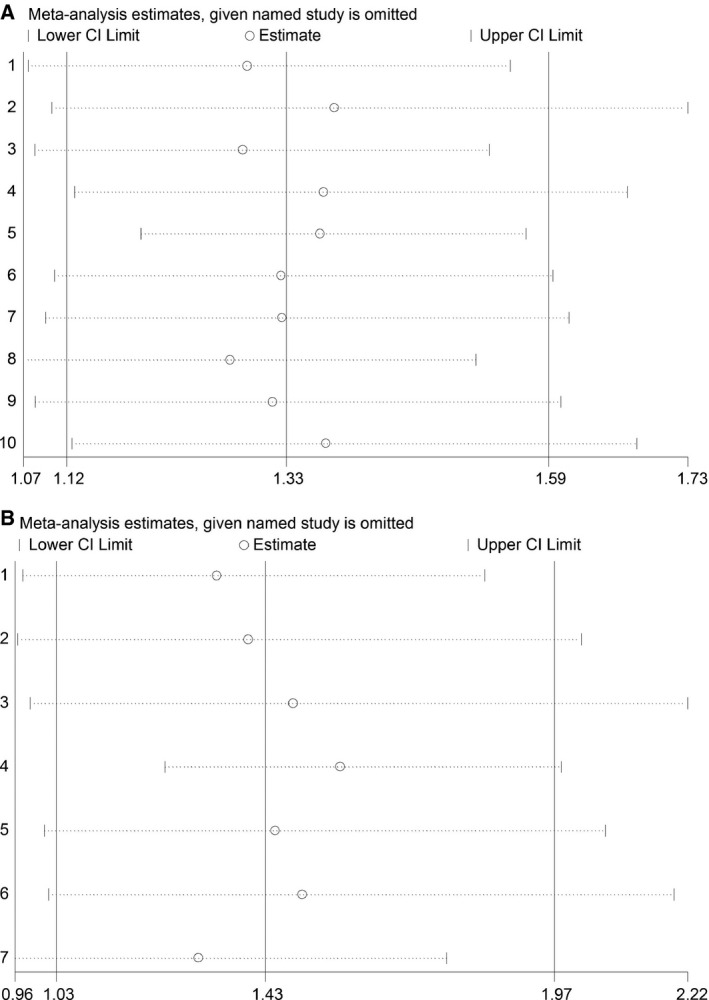
Sensitivity analysis of studies included in this meta‐analysis. (A) OS; (B) DFS.

Sensitivity analysis was used to assess the influence of the each included study on the pooled HR on OS and DFS, and our results showed that the pooled HRs were stable and robust (Fig. 3).

### Publication bias

**Figure 4:**
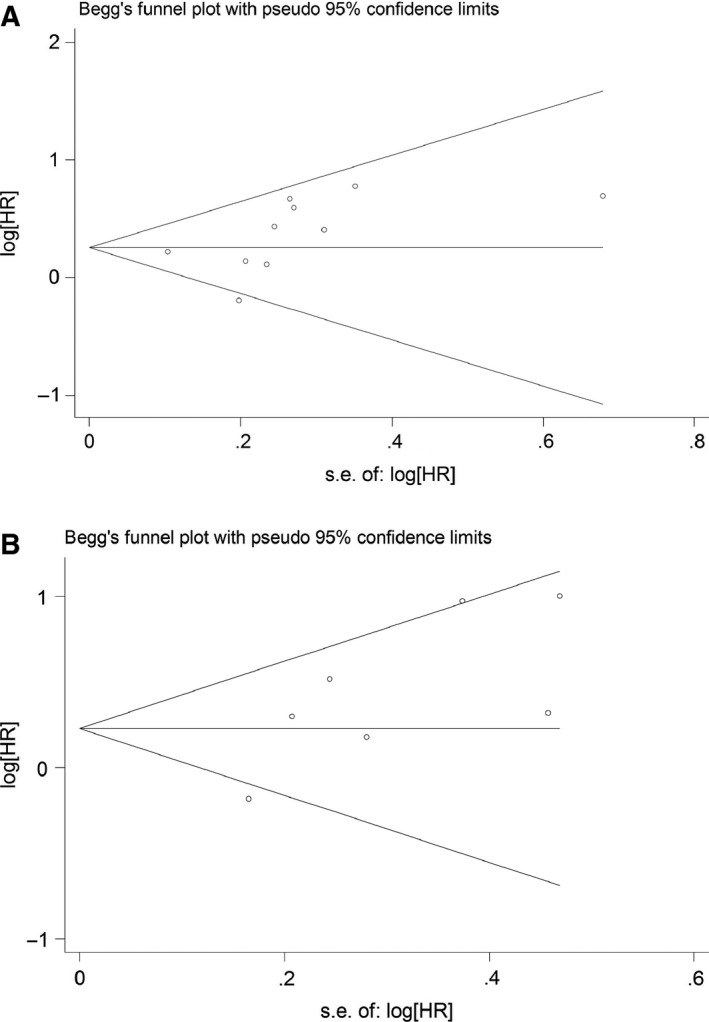
Funnel plot of studies included in this meta‐analysis. (A) OS; (B) DFS.

Begg's test (*P*_B_ = 0.107) and Egger's test (*P*_E_ = 0.162) results showed no evidence of publication bias for OS. Moreover, the shape of funnel plot showed in Fig. 4 supported this conclusion as well. However, Egger's test indicated that there was publication bias in DFS (*P* = 0.044), and the funnel plot showed slightly asymmetry.

## Discussion

In this study, a meta‐analysis containing 12 studies with 3541 patients was conducted to estimate the prognostic effect of PLR on CRC survival, and our study showed that elevated PLR significantly affected OS, DFS, and CSS in overall and Caucasian populations. We also found that elevated PLR was not associated with DFS in CRC patients undergoing surgery alone, but it was associated with poor survival in metastatic patients, which seemed to indicate that there were significant associations between elevated PLR and OS, DFS, and progression‐free survival (PFS) in the metastatic subgroup. Our observation that elevated PLR was significantly associated with poor OS and DFS in metastatic patients will need to be confirmed in further studies, as none of the enrolled studies reported on the relationship between PLR and PFS. However, our findings indicated that elevated PLR is a promising prognostic biomarker for CRC, especially in metastatic Caucasian CRC patients.

Persistent infections and inflammatory responses contribute to 15–20% of cancer‐related deaths worldwide 3 and inflammation is an important part of cancer progression. Lymphocyte, a member of inflammatory cells, taking part in systematic inflammatory response, has been proved to be significantly associated with the survival of various cancers 34, 35, 36, 37, 38, including CRC. Meanwhile, platelet count also was a promising prognostic biomarker for many cancer types 39, 40. Thus, PLR, the ratio of platelet to lymphocyte, may act as a prognostic biomarker in CRC. So, for our study, it is the first study to comprehensively estimate the association between PLR and survival of CRC patients. And the results showed that the PLR was strongly associated with OS, DFS, and CSS of CRC, indicating that elevated PLR could be a promising prognostic biomarker for CRC. At the same time, our result on the relationship between PLR and OS was consistent with the results of the previous meta‐analysis 41, 42, in which fewer than five of CRC relative articles were included and neither DFS nor CSS were reported.

The following reasons may explain our findings. On one hand, lymphocyte, a kind of leukocyte which played a great role in adaptive immune responses, could be recruited from peripheral circulation system to tumor tissues after chronic inflammation and then activated transcription factor of inflammatory cell and tumor cell, such as NF‐ΚB, STAT3, and H1F1α, to promote the production of inflammatory mediators including chemokine and cytokines, such as IL‐6 which is mainly released by CD4 + T lymphocyte 3. Moreover, elevated IL‐6 had been observed to be of great significance in CRC 43. Furthermore, cytokines activated the key inflammatory mediators as well, resulting in more inflammatory mediators being produced. Because of this function of magnification, tumor microenvironments were generated 3, 44, lymphocyte infiltration increased, peripheral lymphocyte decreased, and thus malignant cell escaped from immune surveillance. As a result, it promoted malignant cell to proliferate, infiltrate, and undergo metastasis. On the other hand, platelets, also a major component of peripheral blood, could secrete inflammatory mediators and growth factors, such as VEGF, TNF‐α, and TXA2, which were linked with processes of hemostasis, inflammation, and tissue repair 45. As a result, cancer‐related inflammation made great contributions to the up‐regulation of the ratio of platelet to lymphocyte. Meanwhile, elevated PLR also promoted the CRC progression, leading to a poor survival of CRC patients.

However, some limitations should be addressed as following: first, the summarized data were used in our study, not individual data; second, the outcome of pooled studies were slightly related to PLR and some pooled results were from univariate analysis rather than multivariate analysis; third, the evidence of publication bias was found in DFS.

In conclusion, PLR, an easy and high efficient laboratory biomarker, was closely associated with the survival outcome of CRC, and elevated PLR is a promising prognostic biomarker for CRC, especially in metastatic Caucasian CRC patients.

## Author contributions

BSH and YQP designed the study, HXP and KL acquired the studies and recorded the data, HQY checked the results and revised the draft, TX and XXH contributed to doing analysis, and HXP and SKW drafted the paper.
